# The SMART IRB platform: A national resource for IRB review for multisite studies

**DOI:** 10.1017/cts.2019.394

**Published:** 2019-07-29

**Authors:** Nichelle Cobb, Elizabeth Witte, Maria Cervone, Aaron Kirby, Douglas MacFadden, Lee Nadler, Barbara E. Bierer

**Affiliations:** 1 Health Sciences IRBs Office, University of Wisconsin – Madison, Madison, WI, USA; 2 Harvard Catalyst | The Harvard Clinical and Translational Science Center, Harvard Medical School, Boston, MA, USA; 3 Department of Medical Oncology, Dana Farber Cancer Institute, Boston, MA, USA; 4 Department of Medicine, Brigham and Women’s Hospital, Boston, MA, USA

**Keywords:** Institutional review boards, multicenter clinical research, single IRB review, NIH single IRB policy, sIRB, reliance agreement, master IRB agreements, cede review, cooperative agreement, IRB authorization agreement, research ethics, bioethics, human subject protections

## Abstract

Single institutional review board (IRB) review of multisite research increased in frequency over a decade ago with a proliferation of master IRB reliance agreements supporting statewide and regional consortia and disease- and population-specific networks. Although successful, the increasing number of agreements presented significant challenges and illuminated potential benefits of a single, nationwide agreement. Anticipated changes in federal regulations highlighted the need to systematize and simplify IRB reliance. To address these challenges, the NIH National Center for Advancing Translational Sciences funded a project to establish a national IRB reliance network that would support national adoption of single IRB (sIRB) review. The Streamlined, Multisite, Accelerated Resources for Trials (SMART) IRB Platform launched in July 2016 to facilitate dissemination, adoption, and implementation of a collaboratively developed master IRB reliance agreement and supportive tools and resources. More than 580 institutions have joined SMART IRB’s Master Common Reciprocal Institutional Review Board Authorization Agreement and begun using the SMART IRB platform to support sIRB arrangements. Here, we describe the tenets of the agreement and operational benefits and challenges of its use. SMART IRB’s early success affirms the utility of collaborative, flexible, and centralized approaches to supporting sIRB review while highlighting the need for further national harmonization.

## Background

In multisite studies, the burden and expense of conducting multiple, duplicative ethics reviews by several institutional review boards (IRBs) has been cited as a major barrier to research, delaying research conduct without demonstrable benefit to the protection of human subjects [1,2]. Single IRB (sIRB) review of multisite research began to increase more than a decade ago as academic institutions and health care centers developed local IRB reliance agreements [3–5]. Through frequent interactions, consensus building, and expanded adoption, these institutions developed statewide and regional consortia^1^ to reduce administrative burden on researchers and promote further collaboration. At the same time, institutions engaged in recurring and continuing collaborations focusing on a specific disease, condition, or population developed master reliance agreements to decrease the administrative burden of duplicative review for those partnerships. These regional and disease- or population-specific master agreements^2^ allowed institutions to avoid time- and resource-intensive repetitive negotiation of study-specific reliance agreements. Although these reliance agreements and networks proved locally successful, navigating myriad agreements with varied terms and responsibilities, each requiring institutional legal review and approval, presented significant challenges for institutions and investigators and illuminated the potential benefits of a single, master IRB reliance agreement for use across the nation. Anticipated changes in federal regulations that require increased use of sIRB review of multisite research further highlighted the need to systematize and simplify reliance arrangements.

To address these challenges, in September 2014, the National Institutes of Health (NIH) National Center for Advancing Translational Sciences (NCATS) funded a project^3^ to develop a master IRB reliance agreement to support sIRB review of multisite research across the Clinical and Translational Science Awards (CTSA) consortium. This initial project culminated, in April 2016, with the development of a prototype national master IRB reliance agreement informed by input from 115 institutions, including all 62 then-current CTSA hubs [6] in 33 states, as well as public and private universities, academic health care centers, community hospitals, independent IRBs, and NIH agencies. Collaboration and engagement were essential elements of the process; stakeholders provided input on a variety of issues such as minimum insurance coverage, applicability of an institution’s Federalwide Assurance (FWA), indemnification, and performance of Health Insurance Portability and Accountability Act of 1996 (HIPAA) Privacy Board functions. The resulting agreement was not limited to one type of research but rather applicable to a wide range of human subject research (minimal risk and greater than minimal risk; large and small studies; biomedical and social and behavioral; federally, academically, and industry-funded; investigator-initiated and other). Although the agreement could be used for situations when sIRB arrangements are required (e.g., by policy or requirement of participation in a network), it also provided for the voluntary decision to engage in reliance. The agreement delineated the roles and responsibilities for what were termed “Participating Institutions,” as well as for the “Reviewing IRB(s)” and “Relying Institutions” for research covered by the agreement. In addition, complementary standard operating procedures (SOPs) were developed to guide study-specific implementation of the master agreement; these SOPs were to be the default unless other provisions were agreed upon and documented as a condition of reliance.

In July 2016, NCATS funded the Streamlined, Multisite, Accelerated Resources for Trials (SMART) IRB Platform [7] to facilitate dissemination, adoption, and implementation of this consensual national agreement; develop processes, workflows, and tools to enable IRB reliance on a national scale; and support compliance with the NIH Single IRB Policy (effective date: January 25, 2018) [8] as well as other sIRB arrangements. While leadership for this project centered within the CTSAs of Harvard University, University of Wisconsin-Madison, and Dartmouth College, funding provided for the hiring of regional ambassadors, individuals with significant experience working with IRBs and clinical research, both to provide a wide range of input regarding initiatives and priorities and to promote national adoption. Furthermore, CTSA leadership at academic institutions and at NIH championed the development and dissemination of the system. The first phase of the SMART IRB project (from July 2016 to April 2018) focused on (1) helping institutions join the master IRB reliance agreement (known as the SMART IRB Agreement); (2) supporting implementation of the agreement to enable sIRB review for multisite studies; and (3) spearheading a collaborative effort toward nationwide harmonization of policies and processes related to sIRB review. Beginning in July 2018, the second phase of SMART IRB, led by Harvard University and University of Wisconsin-Madison, focused on further expansion of SMART IRB’s reliance network; increased education and training for investigators, study teams, and IRB and Human Research Protection Program (HRPP) professionals; and continued harmonization of policies and processes related to the initiation and conduct of multisite research using sIRB review.

## Methods: Building a National IRB Reliance Network

Traditionally, all institutions involved in a master IRB authorization agreement would sign a single agreement and keep copies of the signed agreement on file. This approach was untenable on a national scale; therefore, a new approach to enable institutional sign-on was developed. SMART IRB’s Master Common Reciprocal Institutional Review Board Authorization Agreement (the SMART IRB Agreement) [9], the foundation of the SMART IRB platform, is a treaty among SMART IRB Participating Institutions: any eligible institution or IRB organization (IORG) may join by executing a “Joinder Agreement”^4^ to the SMART IRB Agreement. The Agreement is a covenant among the signatories themselves; it is not an agreement with one lead institution, the NIH, or any other single entity.^5^ It is a voluntary, umbrella agreement, meaning that Participating Institutions may elect to use – or not use – the SMART IRB Agreement on a study-by-study basis. When they do elect to use the SMART IRB Agreement, they commit not only to adhere to the terms of the Agreement, but also to comply with applicable US laws and regulations. Thus, the SMART IRB Agreement cannot be used for studies for which local IRB review is required by law but can be used by Participating Institutions outside the USA that wish to review for or rely on US signatories; the agreement, however, is silent on ex-US laws and regulations.^6^

By joining SMART IRB, Participating Institutions eliminate the need to review, negotiate, and sign a new IRB reliance agreement for each study, a review that is often a lengthy process involving institutional legal counsel and requiring the signature of the appropriate institutional official. For each study, Participating Institutions need only document use of the SMART IRB Agreement, the specific reliance arrangements (i.e., Reviewing IRB and Relying Institutions), and any conditions specific to the oversight of that study. The SMART IRB Agreement provides for a certain amount of flexibility in the implementation of study-specific reliance arrangements. Three key areas of flexibility are: (1) who will serve as the Privacy Board for institutions when the HIPAA Privacy Rule applies to the study, with the default expectation being that the Reviewing IRB will serve in this capacity; (2) whether the Reviewing IRB will require a combined or separate approach to HIPAA authorizations, with the default being a combined consent and HIPAA authorization form; and (3) whether the Reviewing IRB will be solely responsible for reporting to federal agencies and sponsors, such as in the case of a finding of an unanticipated problem or serious and/or continuing noncompliance, or will provide a joint report, or delegate the reporting to the affected Relying Institutions, with the default being sole reporting by the Reviewing IRB. SMART IRB has developed a template implementation checklist to assist institutions in documenting how these flexible terms will be implemented for a given study [10].

Institutions join the SMART IRB Agreement individually. In developing the eligibility criteria, consideration was given to how best to build trust between Participating Institutions and promote the protection of human subjects. To be eligible to join the Agreement, an institution must: (1) have an active FWA^7^ (or be an IRB organization) and provide institutional oversight of its human subjects research; (2) have undergone or initiated a quality assessment of its HRPP within 5 years prior to joining^8^; this may be accomplished through accreditation by an external organization (e.g., Association for the Accreditation of Human Research Protection Programs [AAHRPP]) or a proxy (e.g., Office of Human Research Protections [OHRPs] Self-Assessment [11], an internal or external review or audit, or other substantial equivalent); and (3) establish a point of contact (POC) responsible for initial and ongoing implementation and communication regarding the SMART IRB Agreement. An institution’s POC is often, but not always, associated with the IRB or HRPP; some Participating Institutions do not have an IRB or choose to designate a POC from outside the IRB office. Institutions may also designate an Alternate POC to support these functions.

The rationale for requiring an FWA was that maintenance of an FWA indicates an institution’s commitment to human research protections and its accountability for the compliance of its HRPP with federal standards. IRB organizations (IORGs) do not need an FWA; however, these organizations must register with the OHRP and thus demonstrate their knowledge of and compliance with US federal regulations. The status of a Participating Institution’s FWA or IORG registration may be verified via the OHRP website (https://ohrp.cit.nih.gov/search/fwasearch.aspx?styp=bsc).^9^ Reporting to OHRP or other agencies (e.g., unanticipated problems, serious or continuing noncompliance determinations, suspensions, and terminations) is dictated by the institution’s FWA or its policies or by regulation, but nothing in the agreement precludes an institution from reporting to OHRP or another agency. SMART IRB Agreement eligibility sets a baseline expectation for Participating Institutions with IRBs to have processes to assess the quality of their HRPPs, especially IRB function. This requirement was put in place to help build trust between institutions that could potentially serve as Reviewing IRBs for each other. The initial iteration of the SMART IRB Agreement requires all institutions with IRBs to provide information about the quality assessment they perform regardless of whether that institution ever intends to serve as a Reviewing IRB. Significant challenges exist with being able to track which Participating Institutions would serve only as Relying Institutions and any changes that could occur in that designation (e.g., an institution that asserts it will only rely could change to a Reviewing IRB and would thus not be eligible for the Agreement if it had not described how it met the HRPP quality assessment requirement).

Participation in the SMART IRB Agreement does not preclude an institution’s participation in any other IRB reliance agreement or arrangement with any other entity, including with other institutions that are also SMART IRB Participating Institutions. Although institutions may switch to the SMART IRB Agreement to cover (and sunset) their existing reliance relationships, or those going forward, there is no requirement to do so. However, institutions must communicate with one another in advance of ceding review for any research study to clarify which agreement they are using and document the reliance for that research.

The SMART IRB Agreement includes some default requirements that the Reviewing IRB may agree to waive. For example, when using the SMART IRB Agreement, the default requirement is that all Participating Institutions (1) maintain, implement, or have access to a human subjects research quality assurance/quality improvement process, function, program, or service that can conduct and report to the Participating Institution the results of for-cause and not-for-cause audits and (2) maintain sufficient insurance coverage – or self-funded liability coverage in the case of state institutions – to cover the research activities related to a given reliance arrangement. Institutions are encouraged to use the SMART IRB SOPs [12] for studies using the SMART IRB Agreement, but the Reviewing IRB may opt to use its own or other policies and procedures for a reliance relationship, so long as doing so is documented and does not render the Participating Institutions in violation of any term of the SMART IRB Agreement. Participating Institutions must communicate with one another regarding whether the SMART IRB SOPs or another set of policies and procedures will apply to a specific reliance arrangement.

## Responsibilities of the Reviewing IRB

Under the SMART IRB Agreement, a Reviewing IRB^10^ is responsible for overseeing a study from its inception to study closeout, including initial reviews, reportable events, personnel changes, continuing reviews, audits (which it can delegate), and study-wide and local amendments (see Table 1.) The Reviewing IRB must make appropriate records available to a Relying Institution upon reasonable request.

**Table 1.** Roles and responsibilities of the reviewing IRB and relying institutions under the SMART IRB agreement

	Reviewing IRB(s)/Reviewing IRB Institution(s)^1^	Relying Institution(s)^2^
**IRB Registration**	Maintain current IRB registration with OHRP.	Not Applicable
**IRB Membership**	Maintain IRB membership that satisfies requirements of federal policy and other applicable regulations/policies.	Not Applicable
**Policies and Procedures**	Make policies and procedures available to the Relying Institution(s), when applicable and upon request.	Not Applicable
**IRB Review and Oversight**	Perform initial and continuing reviews, and reviews of amendments, unanticipated problems that may involve risks to subjects or others, and potential noncompliance, in accordance with the requirements of Relying Institution’s(s’) FWA(s) and applicable regulations/policies.	Accept Reviewing IRB’s decisions and requirements and require its Research Personnel to provide information that the Reviewing IRB requires for continuing review.
**Local Considerations**	Consider local requirements communicated by Relying Institution(s).	Communicate to the Reviewing IRB requirements of its FWA and any applicable state or local laws, regulations, institutional policies, standards, or other local factors, including local ancillary reviews that affect the conduct or approval of the research at the Relying Institution.
**Recordkeeping**		Require its Research Personnel to maintain all research records, including informed consent documents and HIPAA authorizations, in accordance with applicable federal, state, and local regulations.
**HIPAA** (Collectively, the Health Insurance Portability and Accountability Act of 1996, the Health Information Technology for Economic and Clinical Health Act of 2009, and their implementing regulations)	Serve as Privacy Board, when a study falls under the HIPAA Privacy Rule; may make alternate arrangements (some/all Relying Institutions perform Privacy Board determinations). Ensure PHI will not be used/disclosed unless written authorization obtained from participants, waiver of alteration of authorization granted, or use of limited data set pursuant to a data use agreement. When authorization required, provide authorization language.	With Reviewing IRB, establish whether separate or combined consent/HIPAA authorization will be used. Provide institution-specific language to the Reviewing IRB Notify Reviewing IRB of specific local requirements and restrictions on use and disclosure of PHI that could prevent Reviewing IRB from approving a request for waiver of authorization for the institution.
**Consent Forms**	Provide Relying Institutions/Site Investigators approved informed consent templates (when required). Permit customization of limited site-specific sections. Provide final, approved consent form(s) to Relying Institutions/Site Investigators (directly or via designee, e.g., Lead Study Team).	Provide Reviewing IRB with site-specific information requested/identified in the customizable sections of the Reviewing IRB’s consent form.
**COI**	Consider Relying Institution’s determinations and management plans. Incorporate management plan into deliberations, as applicable. May impose additional prohibitions or requirements more stringent or restrictive than proposed by a Relying Institution. Will not modify plan/mandated disclosure to subjects without discussion with and acceptance by Relying Institution.	Maintain and share COI policies. Perform COI analysis (unless alternate arrangement agreed upon). Communicate COI determinations to Reviewing IRB. Abide by Reviewing IRB COI determinations.
**IRB Decisions, Changes, Lapses in Approval**	Promptly notify Overall PI, Site Investigator(s), and Relying Institution(s) of determinations; review decisions; changes; lapses in approval and applicable corrective action plans.	May not initiate any Research or change to the Research, except where necessary to eliminate apparent immediate hazards to subjects, without Reviewing IRB’s prior approval.
**Unanticipated Problems, Injuries, Complaints**	Promptly notify overall PI, Site Investigators, and Relying Institution(s) about findings of and actions related to: Unanticipated problems involving risks to subjects or others. Subject injuries related to research participation. Significant subject complaints.	Require Site Investigator(s) to promptly notify Reviewing IRB of unanticipated problems that may involve risks to subjects or others, or any subject injuries related to research participation, or any significant subject complaints at the Relying Institution.
**Injury Coverage**	Not Applicable	Ensure provisions of any applicable grant or contract that address financial coverage for research-related injuries in connection with research funded in whole or in part by a non-federal entity are consistent with the approved Research protocol and consent form or that approved protocol and consent form, if more protective of human subjects, will control.
**Complaints**	Not Applicable	Ensure mechanism exists by which local research participants or others may communicate complaints about the research to a local contact.
**Noncompliance, Suspension/Termination of Approval, Restriction/Suspension of Authority**	Promptly notify overall PI, Site Investigators, and Relying Institution(s) about findings of and actions related to: Apparent serious and/or continuing noncompliance Serious and/or continuing noncompliance, including any steps it deems necessary for remediation at the Relying Institution Suspension or termination of IRB approval.	Promptly notify Reviewing IRB of potential noncompliance with applicable regulations or with the IRB’s requirements/determinations, and of any suspension/restriction of its research personnel’s authority to conduct the research.
**Audits, Investigations; Corrective Actions**	May choose to: Conduct audits of the research; Request Relying Institution conduct audit/investigation and report its findings; OR Work with Relying Institution to conduct an audit/investigation. If conducting audit or investigation, promptly notify and report findings of fact to Relying Institution and inform of any corrective actions.	Cooperate with and require its Research Personnel to cooperate with any audit or investigation by the Reviewing IRB/Institution. If asked to do so, conduct own audit/investigation or work cooperatively with the Reviewing IRB/Institution to conduct audit/investigation and report back findings of fact to Reviewing IRB/Institution within a reasonable time frame. Comply with and require its Research Personnel to comply with all corrective actions required by the Reviewing IRB/Institution; may adopt more stringent additional corrective actions.
**Reporting**	Notify Relying Institution if report is required to a regulatory agency, sponsor, funding agency, and/or other oversight authority. Typically, draft report and provide involved Relying Institution(s) opportunity to review before sending to external recipients. Not obligated to adopt comments of a Relying Institution.	Promptly provide any comments on draft report. If requested, promptly prepare draft report and provide Reviewing IRB/Institution with opportunity to review and comment. If making own additional report, provide copy to Reviewing IRB/Institution.
**Communications with Regulatory Agencies**	Promptly notify Relying Institution(s) of any communications received from the FDA, OHRP, and/or other regulatory agencies regarding: Unanticipated problems Suspension or termination of IRB approval Serious and/or continuing noncompliance Other regulatory compliance concerns the Research.	Promptly notify Reviewing IRB/Institution of communications received by or between Relying Institution and FDA, OHRP, and/or other regulatory agencies regarding unanticipated problems, noncompliance, or other compliance concerns regarding the Research. Require Overall PI/Site Investigator(s) to do the same.
**Congruence of Federal Grant Applications/ Contract Proposals** ^**3**^	Review congruence of any federal grant application or contract proposal with the research submitted for review, when required by federal regulations or oversight agencies (unless other arrangements are made).	Not Applicable

IRB, institutional review board; SMART, Streamlined, Multisite, Accelerated Resources for Trials; OHRP, Office of Human Research Protections; FWA, Federalwide Assurance; PHI, protected health information; COI, conflict of interest; PI, Principal Investigator; FDA, Food and Drug Administration;; HIPAA, collectively, the Health Insurance Portability and Accountability Act of 1996, the Health Information Technology for Economic and Clinical Health Act of 2009, and their implementing regulations

1 A Reviewing IRB is the “IRB of record” (including an IRB Organization) to which authority for IRB review and oversight has been ceded by another Participating Institution for an instance of Research under the Agreement. A Reviewing IRB Institution is the institution whose IRB has become the Reviewing IRB for another Participating Institution for an instance of Research under the Agreement.

2 A Relying Institution is a Participating Institution that cedes IRB review to a Reviewing IRB for an instance of Research under the Agreement.

3 IRB review for congruence is no longer required by the Common Rule regulations (2019), and the SMART IRB Agreement allows for this flexibility.

In executing its responsibilities and in performing its review and oversight, the Reviewing IRB must consider local requirements that have been communicated by a Relying Institution. These might include: (1) applicable state or local laws, regulations, institutional policies, standards, or other local factors, including ancillary reviews, relevant to the research that would affect the conduct or approval of the research at the Relying Institution; (2) site-specific information requested/identified in the customizable sections of the Reviewing IRB’s consent form; (3) conflict of interest (COI) determinations, prohibitions, and management plans; and (4) local requirements and restrictions on use and disclosure of protected health information (PHI) that could prevent the Reviewing IRB from approving a request for waiver or alteration of HIPAA authorization with respect to the Relying Institution.

The Reviewing IRB ensures any COI management plan is incorporated into its initial review or other deliberations, as applicable, such as including disclosures to subjects in informed consent forms, and may impose additional prohibitions or conflict management requirements more stringent or restrictive than those proposed by a Relying Institution. However, a Reviewing IRB may not modify or change any management plan or mandated disclosure to participants without discussion with and acceptance by the Relying Institution.

The Reviewing IRB makes its policies and procedures available to Relying Institutions, when applicable and upon request. When informed consent is required, the Reviewing IRB provides approved informed consent templates to the Relying Institutions and the Site Investigators (either directly or through a designee), permitting customization of limited site-specific sections (e.g., availability of treatment/compensation for research-related injury, payment or reimbursement of research costs incurred by subjects, local contacts). There is flexibility in how Reviewing IRBs communicate institutional requirements for consent forms to the Reviewing IRB [13]. The Reviewing IRB reviews and approves the final consent form, including all customized sections, and provides that the final approved consent form to the Relying Institutions and its Site Investigators through the communication mechanism established by the Reviewing IRB.

The Reviewing IRB promptly notifies the Overall Principal Investigator (PI), Site Investigator(s), and the Relying Institutions of determinations (e.g., serious or continuing noncompliance), review decisions (e.g., approval, disapproval, required modifications), and lapses in IRB approval and any applicable corrective action plans. The Reviewing IRB also promptly notifies the Overall PI, Site Investigator(s), and Relying Institution(s) about findings of and actions related to apparent or actual serious or continuing noncompliance, including any steps necessary for remediation of the noncompliance at the Relying Institution; unanticipated problems involving risks to subjects or others; subject injuries related to research participation; significant subject complaints (e.g., those that could affect the conduct of the research); and suspension or termination of IRB approval of the research.

A Reviewing IRB can conduct audits of the research, request a Relying Institution to conduct an audit or investigation and report back its findings, or work cooperatively with a Relying Institution to conduct an audit or investigation. The Reviewing IRB notifies a Relying Institution in advance if it determines that reporting to a regulatory agency, sponsor, funding agency, and/or other oversight authority is required. Typically, the Reviewing IRB will draft the report and provide the involved Relying Institution(s) the opportunity to review the draft report before sending to external recipients, with a minimum of five business days for review; the Reviewing IRB is under no obligation to adopt comments of a Relying Institution. The involved Participating Institutions may agree on an alternate arrangement, whereby the Relying Institution drafts and makes the report or the Reviewing IRB and Relying Institution jointly develop the report. The Reviewing IRB will also promptly notify the Relying Institutions of any communications received from the FDA, OHRP, and/or other regulatory agencies relevant to the ceded research.

## Responsibilities of Relying Institutions

While a Relying Institution cedes IRB oversight of a study, the institution retains responsibility for the protection of human subjects; for compliance with applicable laws, regulations, and ethical standards; and for compliance with the terms of its FWA (see Table 1). Relying Institutions are responsible for ensuring that their local study teams (1) do not initiate a study or any protocol changes – except those to eliminate an apparent immediate hazard – without approval from the Reviewing IRB; (2) provide the Reviewing IRB with information about local study conduct for continuing review; (3) maintain research records (e.g., consent forms, HIPAA authorization); and (4) notify the Reviewing IRB of unanticipated problems, potential noncompliance, and suspension or restriction of study team personnel’s authority to conduct the study.

A Relying Institution must provide information or documentation to a Reviewing IRB, as requested, regarding its research personnel’s education, training, and qualifications, and must also communicate relevant local context (e.g., state and local laws and regulations, institutional policies, local factors, and ancillary reviews) that would affect the conduct or approval of the research at the Relying Institution. A Relying Institution will also provide site-specific information in the customizable sections of the Reviewing IRB’s consent form. Relying Institutions must maintain and share COI policies, and, unless an alternative arrangement is made, perform COI analyses, communicate its COI determinations to the Reviewing IRB, and abide by the Reviewing IRB’s COI determinations.

With regard to the HIPAA Privacy Rule, Relying Institutions work with the Reviewing IRB to establish whether a separate authorization form or combined consent/authorization will be used for the research, provide any institution-specific language, and notify the Reviewing IRB of any specific local requirements and restrictions on use and disclosure of PHI that could prevent the Reviewing IRB from approving a request for a waiver or alteration of HIPAA authorization for the Relying Institution.

A Relying Institution must have an institutional mechanism by which local research participants or others may convey complaints about the research to a local contact and must ensure that the approved protocol and consent form address financial coverage for research-related injury and be able, either through applicable grant, contract, or other arrangements, to uphold the coverage commitments.

If the Reviewing IRB requests an audit or investigation, a Relying Institution must provide research records and related information, meet with representatives from the Reviewing IRB, report any of its own findings to the Reviewing IRB within a reasonable time frame, and help carry out and comply with all corrective actions required by the Reviewing IRB. When reporting to regulatory agencies is required, Relying Institutions must promptly provide any comments on the draft report of the Reviewing IRB or, if requested, promptly prepare the draft report and provide the Reviewing IRB an opportunity to review and comment. As noted above, the involved institutions may agree on an alternate arrangement, whereby the Relying Institution drafts and makes the report or the Reviewing IRB and Relying Institution jointly make the report. A Relying Institution may elect to make its own additional report, in which case, it must provide a copy to the Reviewing IRB. Relying Institutions must promptly notify the Reviewing IRB of any reporting-related communications received from regulatory agencies.

## Adoption and Use of SMART IRB

As noted above, when the advent of a national IRB authorization agreement eliminated the need to negotiate and sign study-specific agreements, a mechanism was required to identify which institutions qualify to join and have agreed to the terms of the master agreement and to allow institutions to attest to using the agreement on a study-by-study basis.

**Fig. 1.**
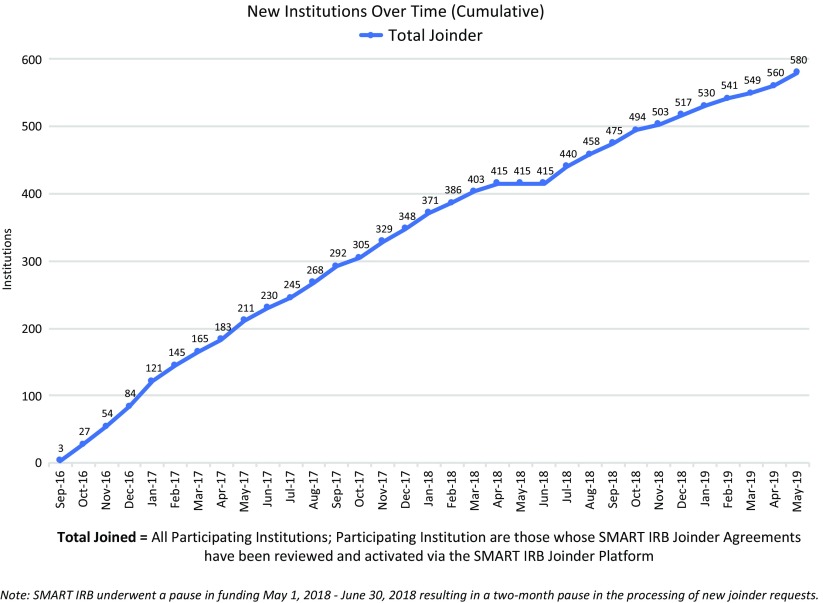
Growth in SMART IRB Participating Institutions over time through May 31, 2019. IRB, Institutional Review Board; SMART, Streamlined, Multisite, Accelerated Resources for Trials

Institutions “join” the SMART IRB Agreement via an online platform rather than through the traditional exchange between institutions of a signed agreement that covers ceded research. A Joinder platform was developed and launched in September 2016 to allow Participating Institutions to sign onto the SMART IRB Agreement. The Joinder platform collects key information (e.g., institution name and address; FWA# or IORG#; whether the institution maintains an IRB and, if so, information about the process by which an institution satisfies the requirement regarding the quality assessment of its HRPP and IRB; names and contact information for the designated POC(s), notices; and the institutional official (IO)) to generate an institution-specific Joinder Agreement to the SMART IRB Agreement; the Joinder platform allows SMART IRB personnel^11^ to review and activate a submitted joinder agreement, and catalogs all SMART IRB Participating Institutions to populate a list of signatories on the SMART IRB website (https://smartirb.org/participating-institutions/). Within the first 4 months, over 100 institutions joined SMART IRB, including all 62 then-current CTSA program hubs; as of May 31, 2019, more than 580 institutions had joined, including universities, academic medical centers, community hospitals, cancer centers, patient-powered research networks, and independent/commercial IRBs (see Fig. 1.)

Sign-on was encouraged by the SMART IRB team deploying a team of regional ambassadors to reach out to institutions about joining the Agreement and answer questions about the terms of the Agreement and process for joining. SMART IRB ambassadors are IRB experts and HRPP professionals from across the country, who provide guidance on eligibility criteria, assist institution in the process of joining, and provide local training and support to enable sign-on to and implementation of the SMART IRB Agreement. Regional ambassadors were joined in their efforts by representatives from the National Patient-Centered Clinical Research Network and the Research Centers in Minority Institutions Translational Research Network, and the ambassador team as a whole has been key to the dissemination and adoption of the SMART IRB Agreement. Over time, some ambassadors have taken on broader roles, providing support in education and other key focus areas.

In addition to facilitating sign-on to and implementation of the Agreement, Ambassadors also support the creation, testing, dissemination, and adoption of tools and resources to aid institutions in the implementation of sIRB review. Recognizing that institutions and investigators vary significantly in their experience with, and established infrastructure and resources to support, sIRB review, the SMART IRB team has developed resources that target three key roles in a sIRB arrangement: study teams, Reviewing IRBs, and Relying Institutions. Resources included: template for documenting reliance arrangements; a checklist to allow institutions to document how they implement the flexible provisions of the SMART IRB Agreement; communication plan for sIRB review, which documents key communication roles; template description of SMART IRB and template IRB letter of support for grant applications; local context survey; and investigator responsibilities checklists. All resources are freely available at https://smartirb.org/resources/.

Additionally, to provide support related to documenting study-specific reliance arrangements, establishing a Reviewing IRB, and facilitating communication between institutions, a central, workflow-based, online platform termed the SMART IRB Online Reliance System (ORS) was developed. The system provides a mechanism for investigators and IRB/HRPP and other institutional administrators to request, track, and document study-specific reliance arrangements under the Agreement. ORS provides research teams with a standard mechanism to initiate sIRB requests, ensuring that the investigator’s home institution is aware of the reliance request, and automatically notifying designated representatives from all engaged sites so that they may collaborate efficiently in determining suitable arrangements. The system identifies for investigators the information and documents needed by the institutional representatives to consider reliance arrangements and, upon submission of a request, ensures this information is routed to the appropriate parties. ORS uses tailored informatics workflows to maximize communication and coordination throughout the reliance determination process. In the case of Reviewing IRBs and Relying Institutions, the ORS provides a robust communication platform that allows the institutions – with security and specificity – to track the status and outcome of reliance requests, thus ensuring compliance with federal regulations governing sIRB review.

**Fig. 2.**
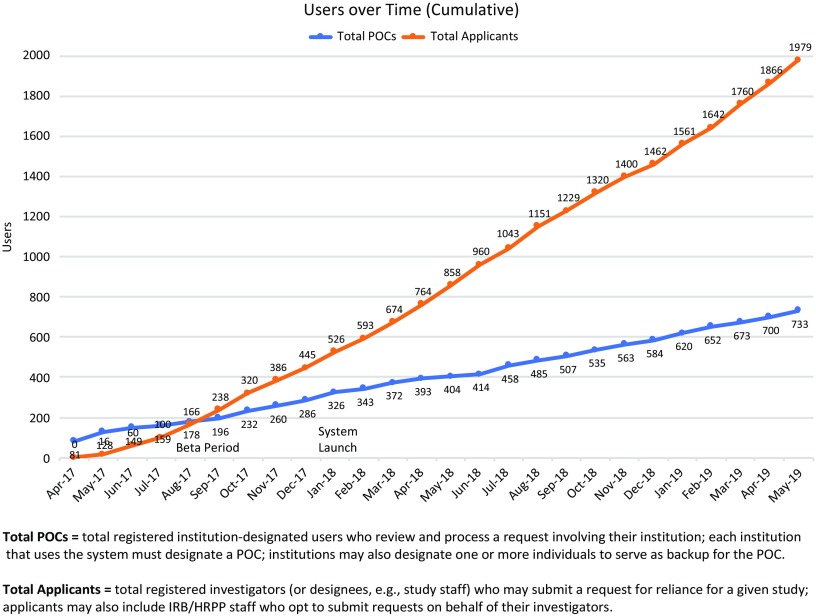
Growth in Online Reliance System users through May 31, 2019. POC, Point of Contact; IRB, Institutional Review Board; HRPP, Human Research Protection Program

**Fig. 3.**
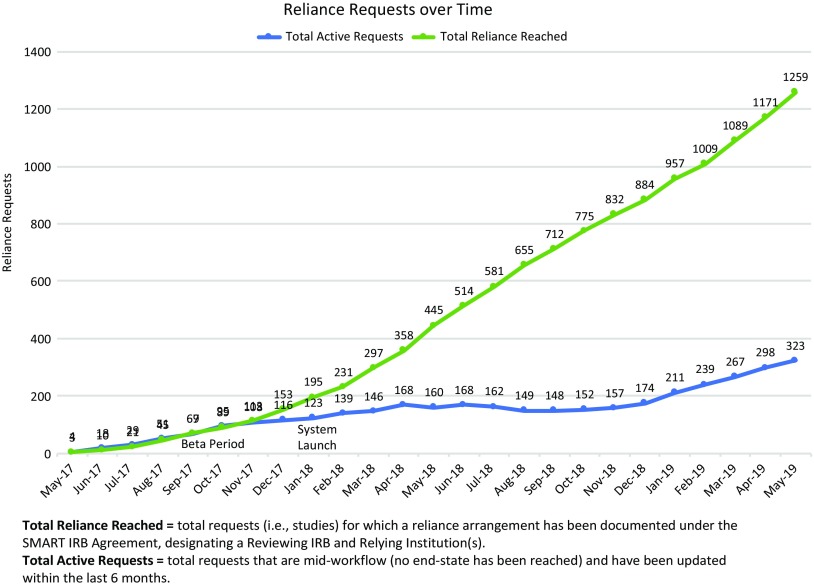
Cumulative Online Reliance System arrangements through May 31, 2019. IRB, Institutional Review Board; SMART, Streamlined, Multisite, Accelerated Resources for Trials

First launched in beta^12^ in May of 2017, the ORS drew early adopters whose feedback informed and helped prioritize ongoing system development. The system fully launched in January 2018, and as of May 31, 2019, had well over 2700 registered users (see Fig. 2); in that time period users have submitted more than 1600 requests for reliance, and more than 1250 studies have documented reliance arrangements in the ORS (see Fig. 3). While the use of the system is not required for studies using the SMART IRB Agreement, the system is a free resource available to help all who have joined SMART IRB streamline the coordination and documentation of study-specific reliance arrangements.

## Supporting Single IRB Review

The ORS and other supportive resources may be accessed via SMART IRB’s open-access online compendium, which provides frequently asked questions (FAQs), best-practice templates, checklists, guidance, and other tools to aid in implementation of sIRB review. Users can filter resources for a specific role (i.e., study teams, reviewing IRBs, relying institutions, IRB/HRPP staff) or topic (i.e., joining SMART IRB, setting up reliance, implementing the Agreement, preparing funding applications). Periodic webinars help institutions get started with SMART IRB and the ORS, implement the SMART IRB Agreement, understand the roles and responsibilities of a Relying Institution or a Reviewing IRB, and introduce how and when to use SMART IRB’s available resources. Recordings of past webinars are available to provide users just-in-time information to support their reliance needs. Ambassadors and SMART IRB team members have attended regional and national events to present and answer questions about SMART IRB specifically and sIRB review more generally. In April 2019, SMART IRB hosted an sIRB boot camp for IRB and HRPP professionals to help these individuals and their institutions successfully implement sIRB review by leveraging the SMART IRB Agreement and supportive resources. This day-long session was attended by more than 100 regulatory professionals from 21 universities, medical centers and hospitals, private companies, and organizations throughout the USA, including the NIH.

As institutions increasingly use sIRB review of multisite research protocols, inconsistencies among local policies and processes relevant to IRB review and the conduct of research have become apparent. Some variation reflects institutional differences in contracting, such as different policies and approaches to indemnification; these differences do not touch the participant or the study team, and while inconvenient and a cause of delay do not impact research conduct. However, variations in institutional policies, requirements, and procedures that impact the conduct of the research, the expectations of the investigators and their teams, and the treatment of the participant make it challenging for a well-meaning study coordinator or investigator to remember and comply with the specifics for each study. These variations are often trivial, such as the age of assent being 12 or 13, or the days allowed for reporting of an unanticipated problem; compliance with the requirements of the Reviewing IRB may render the investigator noncompliant with the policies of their own Relying Institution. Disparate local policies and differing SOPs frustrate – and unnecessarily burden – those who are charged with implementing single site review of multisite studies.

To begin the long process of institutional alignment, the SMART IRB team and NCATS convened the Harmonization Steering Committee (HSC) composed of a broad array of stakeholders, including representatives from NIH, OHRP, Food and Drug Administration (FDA), Veterans Affairs (VA), AAHRPP, Federal Demonstration Partnership, NCATS Trial Innovation Network, and independent and institutional IRB and HRPP professionals, to develop, demonstrate, and disseminate unified policies, processes, and procedures for sIRB review. Meeting monthly, the HSC focuses on standardizing methodologies, processes, and infrastructure critical to support multisite studies. Initially, five working groups, each with 8–9 members from academia, independent IRBs, government, and industry, were charged with addressing: (1) institutional and local/state responsibilities, (2) institutional versus IRB responsibilities, (3) sIRB fees and charging models, (4) reportable events, and (5) standard consent templates. Building upon current practices, working groups sought to define common elements, identify differences, and aggregate successful workflows; each deliverable was reviewed by the HSC, posted for public comment for a period of 45 days, amended, revised, and finalized. As of May 31, 2019, five harmonized policies or templates are available for broad adoption: (1) Fees and Costing Models under NIH sIRB Policy, (2) Institution versus IRB Responsibilities Guidance, (3) Institutional Profile, (4) Protocol-Specific Requirements Document, and (5) Reportable Events Recommendations. To access the documents and learn more about the development process, see https://smartirb.org/harmonization/. Current working groups are developing (1) a reciprocal, comprehensive, harmonized indemnification agreement; (2) additional FAQs to address HIPAA issues that arise in reliance agreements; and (3) recommendations on processes for and communication of changing investigators and their study staff after study approval.

## Discussion: Challenges and Next Steps

In response to external pressures catalyzing and now mandating the shift to sIRB review, SMART IRB has sought to connect IRBs, HRPPs, and research administration professionals to provide a sounding board for questions, to address challenges, and to communicate best practices. Starting with the development of the SMART IRB Agreement, SMART IRB has relied upon a wide range of stakeholders to ensure that its resources reflect and support the breadth and depth of multisite research. Indeed, the active engagement and support of CTSA leadership, institutional officials, human research professionals, and IRB administrators throughout the USA made this work possible. Through established outreach efforts and collaboration, SMART IRB is poised to facilitate nationwide adaptation to changes in policy and regulations or other changing research needs.

Looking ahead, the incipient changes in the Final Common Rule will require revision of the SMART IRB Agreement, revisions that may be significant but that nevertheless afford the opportunity to introduce additional changes, should they be recommended by the stakeholder community and deemed essential to the continued growth of the SMART IRB network and use of the SMART IRB Agreement. The SMART IRB team is in the process of soliciting input from representatives of Participating Institutions and from the public; the process is transparent. If it is deemed necessary, there will be time for a facilitated sign-on process to an updated agreement to ensure Participating Institutions are able to transition without a gap in participation.

Pending changes to federal regulations requiring sIRB review for multisite human research studies, scheduled to be effective in 2021, along with the existing, though relatively new, NIH sIRB policy, require institutions to substantially re-engineer their HRPPs, conceiving and implementing changes to infrastructure, policies, and procedures, while educating and reorienting regulatory personnel and study teams. SMART IRB presents opportunities for efficiencies and expedited processes in conducting IRB oversight and review. Since its introduction, the SMART IRB Platform (the Agreement, SOPs, ORS, and other supporting resources) has been widely adopted, with many institutions having retired or in the process of retiring other existing master reliance agreements and fully transitioning to the SMART IRB Agreement. As noted above, the Agreement does not preclude the use of other reliance agreements, and institutions may reasonably determine that continued use of existing agreements may, in certain cases, be preferable to the use of the SMART IRB Agreement (e.g., for studies already using a different agreement). However, it is our expectation that, with the continued growth of the SMART IRB network, the need to develop *new* master agreements has been mitigated and Participating Institutions will determine the appropriate time frame to transition completely to the SMART IRB Agreement. As with the work of the HSC, great benefit is to be found in further alignment of processes across institutions, including the use of a common master reliance agreement. That said, the work of facilitating sIRB review is not done: many institutions and investigators are still learning the impact of IRB reliance on the execution of multisite studies and how local processes and resources must adapt to accommodate the shift in research oversight. SMART IRB has and will continue to support IRBs, institutions, and study teams as they successfully transition to and thrive in sIRB review of multisite research.

## Footnotes

1 E.g., Harvard Catalyst/New England; Ohio Collaborative; University of California Biomedical, Research, Acceleration, Integration, & Development (UC BRAID); University of New Mexico; University of Texas; University of Wisconsin–Madison; Vanderbilt University.

2 E.g., PEDSnet, The Network for Excellence in Neuroscience Clinical Trials (NeuroNEXT), National Institutes for Health (NIH) StrokeNet, National Cancer Institute (NCI) Central Institutional Review Board, Veterans Affairs (VA) Central IRB, Rare Diseases Clinical Research Network.

3 Led by the Dartmouth, Harvard, and Wisconsin-Madison CTSAs from September 2014 to April 2016; IRBrely was funded by the NIH National Center for Advancing Translational Sciences (NCATS) through its Clinical and Translational Science Awards (CTSA) Program, grant number 3UL1TR001086-02S2.

4 The agreement by which an institution represents and warrants that it meets all eligibility requirements for participation in the SMART IRB Agreement and agrees to be bound by the terms and conditions of that Agreement.

5 While the SMART IRB Agreement is an agreement between and among the Participating Institutions, a central SMART IRB team provides and supports the essential infrastructure to facilitate institutions’ joining and use of the SMART IRB Agreement and related resources.

6 The SMART IRB Agreement is forward-looking in that it does not preclude ex-US signatories. That said, currently only US institutions participate; any non-US institutions wishing to join could not do so via the Joinder System. Further modifications may be necessary to the Agreement.

7 Any institution that is a separate legal entity under another institution’s FWA or that has its own FWA or IRB organization number must execute its own joinder agreement, regardless of ongoing or legal affiliations with other Participating Institutions.

8 This assessment is currently required only if the institution maintains an IRB or is an IRB organization.

9 Questions regarding whether a given entity is included within a specific FWA should be addressed by the designated Points of Contact, particularly when name of the engaged institution differs from that listed on the OHRP website.

10 References to the Reviewing IRB in the SMART IRB Agreement also include reference to the “Reviewing IRB Institution,” which is defined as the Participating Institution whose IRB has become the Reviewing IRB for another Participating Institution for any instance of Research under the Agreement. Herein, for ease of reading, we use the term Reviewing IRB to refer to the Reviewing IRB and/or the Reviewing IRB Institution, as applicable.

11 While the SMART IRB Agreement is developed as a treaty with no “lead” institution, some central activities need to be assumed by personnel knowledgeable in the regulatory and policy requirements of single IRB review of multisite trials, as well as the specifics of the SMART IRB Agreement, education, harmonization, the informatics platforms, and website (SMARTIRB.org). These responsibilities are currently executed by SMART IRB leadership and the team of dedicated individuals and ambassadors funded by NCATS and institutional support.

12 A beta release is typically the first time that software is available outside of the organization that developed it. The beta phase of the Online Reliance System provided early access to the SMART IRB community, and the larger user-set allowed system bugs to be identified and resolved as new features and functionality were added.

## References

[1] Menikoff J. The paradoxical problem with multiple IRB review. New England Journal of Medicine 2010; 363: 1591–1593. 20942660 10.1056/NEJMp1005101

[2] Silberman G , Kahn K. Burdens on research imposed by Institutional Review Boards: the state of the evidence and its implication for regulatory reform. Milbank Quarterly 2011; 89(4): 599–627. 22188349 10.1111/j.1468-0009.2011.00644.x PMC3250635

[3] Winkler SJ , et al The Harvard Catalyst Common Reciprocal IRB Reliance Agreement: an innovative approach to multisite IRB review and oversight. Clinical and Translational Science 2015; 8(1): 57–66. doi: 10.1111/cts.12202. 25196592 PMC4329026

[4] Cola PA , Reider C , Strasser JE. Ohio CTSAs implement a reliant IRB model for investigator-initiated multicenter clinical trials. Clinical and Translational Science 2013; 6(3): 176–178. doi: 10.1111/cts.12074. 23751020 PMC3914633

[5] National Institutes of Health National Center for Advancing Translational Sciences (NCATS). *IRB reliance: A new model for accelerating translational science [Internet]* 2014 Retrieved from https://ncats.nih.gov/pubs/features/irb-reliance. Accessed October 1, 2018.

[6] National Institutes of Health National Center for Advancing Translational Sciences (NCATS). *CTSA program hubs [Internet]* 2018 Retrieved from https://ncats.nih.gov/ctsa/about/hubs. Accessed October 18, 2018.

[7] National Institutes of Health National Center for Advancing Translational Sciences (NCATS). *NCATS SMART IRB platform [Internet]* 2016 Retrieved from https://ncats.nih.gov/ctsa/projects/smartirb. Accessed October 18, 2018.

[8] National Institutes of Health. *Final NIH policy on the use of a single institutional review board for multi-site research* NOT-OD-16-094. 2018 Retrieved from https://grants.nih.gov/grants/guide/notice-files/NOT-OD-16-094.html. Accessed October 18, 2018.

[9] Streamlined, Multisite, Accelerated Resources for Trials IRB Reliance platform (SMART IRB). *Master Common Reciprocal Institutional Review Board Authorization Agreement [Internet]* 2016 Retrieved from https://smartirb.org/agreement/. Accessed October 18, 2018.

[10] Streamlined, Multisite, Accelerated Resources for Trials IRB Reliance platform (SMART IRB). *SMART IRB agreement implementation checklist and documentation tool [Internet]* 2018 Retrieved from https://smartirb.org/sites/default/files/SMART_IRB_Agreement_Implementation_Checklist_FORM.pdf. Accessed October 18, 2018.

[11] Health and Human Services Office for Human Research Protections. *OHRP QA self assessment tool [Internet]* 2018 Retrieved from https://www.hhs.gov/ohrp/education-and-outreach/human-research-protection-program-fundamentals/ohrp-self-assessment-tool/index.html. Accessed October 18, 2018.

[12] Streamlined, Multisite, Accelerated Resources for Trials IRB Reliance platform (SMART IRB). *SMART IRB Master Common Reciprocal Institutional Review Board Authorization Agreement Standard Operating Procedures [Internet]* 2016 Retrieved from https://smartirb.org/sites/default/files/SMART_IRB_SOP-090816.pdf. Accessed October 18, 2018.

[13] Streamlined, Multisite, Accelerated Resources for Trials IRB Reliance platform (SMART IRB). *Inserting “Local Context” language in informed consent documents [Internet]* 2017 Retrieved from https://smartirb.org/sites/default/files/Local_Context_Language_Guidelines.pdf. Accessed October 18, 2018.

